# Inoculum Potential of *Fusarium* spp. Relates to Tillage and Straw Management in Norwegian Fields of Spring Oats

**DOI:** 10.3389/fmicb.2016.00556

**Published:** 2016-04-22

**Authors:** Ingerd S. Hofgaard, Till Seehusen, Heidi U. Aamot, Hugh Riley, Jafar Razzaghian, Vinh H. Le, Anne-Grete R. Hjelkrem, Ruth Dill-Macky, Guro Brodal

**Affiliations:** ^1^Division of Biotechnology and Plant Health, Norwegian Institute of Bioeconomy ResearchÅs, Norway; ^2^Department of Plant Pathology, University of MinnesotaSt. Paul, MN, USA

**Keywords:** *Fusarium langsethiae*, spore traps, qPCR, straw residues, *Fusarium graminearum*, *Fusarium avenaceum*

## Abstract

The increased occurrence of *Fusarium*-mycotoxins in Norwegian cereals over the last decade, is thought to be caused by increased inoculum resulting from more cereal residues at the soil surface as a result of reduced tillage practices. In addition, weather conditions have increasingly promoted inoculum development and infection by *Fusarium* species. The objective of this work was to elucidate the influence of different tillage regimes (autumn plowing; autumn harrowing; spring plowing; spring harrowing) on the inoculum potential (IP) and dispersal of *Fusarium* spp. in spring oats. Tillage trials were conducted at two different locations in southeast Norway from 2010 to 2012. Oat residues from the previous year’s crop were collected within a week after sowing for evaluation. IP was calculated as the percentage of residues infested with *Fusarium* spp. multiplied by the proportion of the soil surface covered with residues. *Fusarium avenaceum* and *F. graminearum* were the most common *Fusarium* species recovered from oat residues. The IP of *Fusarium* spp. was significantly lower in plowed plots compared to those that were harrowed. Plowing in either the autumn or spring resulted in a low IP. Harrowing in autumn was more effective in reducing IP than the spring harrowing, and IP levels for the spring harrowed treatments were generally higher than all other tillage treatments examined. Surprisingly low levels of *F. langsethiae* were detected in the residues, although this species is a common pathogen of oat in Norway. The percentage of the residues infested with *F. avenaceum, F. graminearum, F. culmorum*, and *F. langsethiae* generally related to the quantity of DNA of the respective *Fusarium* species determined using quantitative PCR (qPCR). *Fusarium* dispersal, quantified by qPCR analysis of spore trap samples collected at and after heading, generally corresponded to the IP. *Fusarium* dispersal was also observed to increase after rainy periods. Our findings are in line with the general understanding that plowing is a means to reduce the IP of *Fusarium* spp. in cereal fields. The main inoculum source for *F. langsethiae* remains unclear. Our results will be useful in the development of forecasting tools to calculate the risk of *Fusarium* in cereals.

## Introduction

Fusarium head blight (FHB) is an important fungal disease of cereals (Parry et al., 1995). It can cause significant yield losses and reduced grain quality. The disease is caused by several *Fusarium* species, which survive largely in soil and on crop residues. The species abundance in the field is influenced by environmental conditions (Xu et al., 2008). *Fusarium* spp. produce a range of different mycotoxins, and if consumed, contaminated grain can be harmful for animals and humans (Desjardins, 2006).

Soil tillage is important to loosen the soil, to prepare a good seedbed, for the incorporation of plant residues, and to control weeds (Håkansson et al., 1998) and plant diseases (Bockus and Shroyer, 1998). However, due to increased risks from erosion and nutrient runoff from tilled fields, the Norwegian authorities encourage farmers to reduce soil tillage operations. Therefore, primary tillage operations in Norwegian cereal fields are more commonly performed in spring, and reduced tillage operations have become more prevalent (Tørresen et al., 2012). In Scandinavia, no-till systems can be used successfully under a wide range of soil types and weather conditions (Rasmussen, 1999). Reports from long-term trials in Norway, indicate that there is little difference in grain yields between reduced tillage and plowed treatments on loamy soil although average yields may be significantly lower on silt, sandy loam, and some clay soils where reduced tillage practices are implemented (Riley et al., 1994, 2005, 2009, 2014). The use of reduced tillage practices does, however, lower the labor requirement and machinery costs (Riley et al., 1994), and the practice has been demonstrated to be a profitable practice in a German study of a crop rotation systems in wheat (Verch et al., 2009). Thus, under certain conditions, reduced tillage practices may be a good way to ensure sustainable crop production with little negative influence on grain yield.

Over the last 15 years, the average *Fusarium* infection levels of spring cereal seeds in Norway have been more than doubled compared to the previous 30 years, and the infection levels have been positively correlated with July rainfall, which is the flowering month of Norwegian spring cereals (Norwegian Scientific Committee for Food Safety, 2013). The FHB/mycotoxin situation has become a serious challenge for the Norwegian grain industry, especially in oats, which is an important crop in Norway covering approximately 25% of the cereal cultivation acreage.

In a Norwegian survey of oats sampled from 2004 to 2009, the following ranking of *Fusarium* species was made based on the DNA concentrations of the *Fusarium* spp. analyzed (from high to low): *Fusarium graminearum = F. langsethiae = F. avenaceum > F. poae > F. culmorum* (Hofgaard et al., 2016). *F. graminearum* is more prevalent than it has been at any time (Bernhoft et al., 2010; Aamot et al., 2015; Hofgaard et al., 2016). The increase in *Fusarium* spp., particularly *F. graminearum*, is thought to have resulted directly from the increase of cereal crop residues remaining on the soil surface, combined with weather conditions that promote *Fusarium* growth and infection of these cereals that has promoted inoculum survival and production (Norwegian Scientific Committee for Food Safety, 2013). Diseases caused by residue-borne pathogens, including FHB, are reported to increase with increasing amounts of crop residues (Bockus and Shroyer, 1998; Dill-Macky and Jones, 2000). In order to minimize the risk of erosion and nutrient runoff and at the same time ensure suitable grain quality, it is important to identify tillage practices that are suitable for Norwegian conditions but which do not promote the development of residue-borne diseases.

In areas with low *Fusarium* inoculum pressure from surrounding fields, residues from the previous crop are considered an important source of inoculum within a field (McMullen et al., 2012). FHB severity and the mycotoxin contamination of cereals has been reported to be influenced by the type and quantity of previous crop residues (Dill-Macky and Jones, 2000). Similarly, the presence of *Fusarium* spp. in association with plant residues has been demonstrated to vary with the crop species, plant tissue, decomposition stage, soil biota, and microclimate (Champeil et al., 2004a; Pereyra and Dill-Macky, 2008). The pathogenic *Fusarium* species have a temporary advantage as they can colonize host plant tissues ahead of the saprophytic fungi that only colonize plant residues after they are incorporated into the soil (Bruehl and Lai, 1966). *F. graminearum* has been reported to survive for years in crop residues and for longer periods on residues left on the soil surface than on buried residues (Pereyra et al., 2004). Similarly the formation of perithecia and macroconidia has been observed to be reduced if the residues have been buried for some time (Khonga and Sutton, 1988). Residues that are buried decompose more quickly when in contact with the soil with residue decomposition being influenced by temperature and moisture as well as the activity of antagonistic microorganisms (Leplat et al., 2013).

*Fusarium avenaceum, F. culmorum, F. graminearum. F. poae*, and *F. sporotrichioides* are *Fusarium* species often identified on cereal crop residues (Dill-Macky and Jones, 2000; Köhl et al., 2007; Fernandez et al., 2008; Golkari et al., 2008; Pereyra and Dill-Macky, 2008; Postic et al., 2012). In a Canadian study, *Fusarium* spp. were isolated from more than 50% of the cereal residues collected from producers’ fields (Fernandez et al., 2008). The various *Fusarium* species are differentially influenced by environmental conditions. *F. graminearum* is most prevalent where humid and relatively warm conditions prevail whereas *F. avenaceum* and *F. culmorum* is more prevalent under cool and wet or humid conditions (Xu et al., 2008). The infestation of residues by *F. avenaceum* is reported to be more stable over time compared to that of *F. graminearum* (Hogg et al., 2010; Palazzini et al., 2013).

Many studies have focused on the effect of tillage regimes on the subsequent development of *Fusarium* and mycotoxins in cereal grains, mainly wheat (Henriksen, 1999; Dill-Macky and Jones, 2000; Guo et al., 2010; Munger et al., 2014), and plowing is often considered as the best tillage practice to reduce the risk of *Fusarium* disease development in cereals. However, it is sometimes difficult to find a direct link between tillage practices and the occurrence of mycotoxins, as *Fusarium* inoculum may be dispersed aerially over large distances (Lori et al., 2009; Prussin et al., 2014a). Several studies have shown that the development of *Fusarium* and mycotoxins in cereals is related more to the amount of residues than to the tillage regime (Maiorano et al., 2008). Only a few studies have focused on the effect of tillage regimes on the presence of *Fusarium* spp. in crop residues within a specific field (Dill-Macky and Jones, 2000; Munger et al., 2014). In Norway, no studies have been published on the presence of *Fusarium* in cereal residues. Little information is published on the influence of weather conditions on the development and spread of *Fusarium* from cereal crop residues within a field and none of these studies have been conducted in regions where *F. avenaceum* and *F. langsethiae* are among the prevalent pathogens associated with FHB of oat. Such information would be valuable for developing best practices for reducing FHB including models to predict the effect of weather in combination with tillage regime and previous cropping on the development and spread of *Fusarium* species.

The objective of this study was to elucidate the influence of various tillage and straw coverage regimes on the IP of *Fusarium* spp. in spring oats in Norway.

## Materials and Methods

### Field Trials

Two tillage trials with continuously grown oats were conducted at two locations in southeast Norway (Solør and Østfold) over 3 years 2010–2012. The trial at Solør was established on silty soil following a precrop of oat, and the trial at Østfold was established on clay soil following a precrop of winter wheat. The dates of seeding, tillage operations and harvesting are presented in Seehusen (2014).

Each oat trial had a randomized split-plot design with two replicate blocks. The two main residue treatments (plot size 42 m × 15 m) comprised I: most of the residues removed and II: all residues chopped and retained on the field. The main plots were separated by a minimum border of 6 m to allow for the operation of tillage implements. Within each main treatment plot, split-plots (6 m × 15 m) with different tillage regimes were established. Plant material from four of these regimes were used in this study: shallow harrowing (5–6 cm) conducted in spring (SSH), shallow plowing (12–15 cm, furrow plow) conducted in spring (SSP), shallow harrowing (5–6 cm) conducted in autumn (SAH), and deep plowing (25 cm, furrow plow) conducted in autumn (DAP). The type of machinery used varied between locations, but at each location the same implements were used in most years. All trials were harvested with a stubble height of 10–15 cm. The straw was baled and removed from those plots where the treatment called for residues to be removed. In the treatments where the residues were retained, the straw was cut to an average length of 6–7 cm, using a straw cutter mounted on the combine harvester or a stubble chopper, and spread evenly over the whole plot surface. Final seedbed preparation was done by harrowing to <5 cm, before sowing with a combined fertilizer and seed drill and rolling with a Cambridge roller. The location of the plots was fixed throughout the experimental period (2010–2012). The proportion of the soil surface area covered with straw residue was recorded within a week after sowing each year at all locations using the line-transect method which involves the use of a cord with 100 equally spaced knots (Morrison et al., 1993). No fungicide, insecticide or plant growth regulators were used in these trials. Additional details of the various tillage treatments and yield parameters examined are presented in Seehusen (2014).

### Assessment of *Fusarium* on Straw Residues

For assessment of *Fusarium* spp. on residues, straw of oats was collected each year at all field locations within a week of sowing. In 2010, the 1st year of the experiment, residues were collected across the whole field area, in order to calculate the background level of *Fusarium* spp.. In 2011 and 2012, oat straw residues were collected from each treatment. Within each experimental plot, residues were collected from four 1 m × 1 m quadrats outside the area designated to be harvested. The residues were dried at 25°C for 24 h and stored at room temperature until used for the recovery of *Fusarium* spp.

For the recovery of *Fusarium* species, 50 pieces of straw from each plot were analyzed, except from Solør in 2011 where 100 pieces were used. The straw pieces, 1.5–2 cm long and mostly including a node, were surface disinfected in 0.5% NaOCl for 30 s, transferred to 70% alcohol for 15 s, then rinsed three times with sterile distilled H_2_O and then finally transferred to sterile filter paper to remove surface water. The straw pieces were then plated onto Petri dishes containing a modified CZID (Abildgren et al., 1987) in which iprodione was replaced with propiconazole (0.75 mg/l). The plated residues were then incubated for 7–10 days, under alternating 12 h darkness and 12 h near ultra violet light (‘black light’) and white light at 20°C. *Fusarium* mycelium, observed following the incubation period, was transferred to SNA (Nirenberg, 1976) containing chlortetracycline to reduce bacterial growth. A small piece of sterile filter paper was placed on the agar surface to promote sporulation. The SNA cultures were incubated for 10–14 days (incubation conditions as above) and used for the morphological identification of *Fusarium* species (Leslie and Summerell, 2006). The percentage of *Fusarium*-infested straw residues was calculated as the number of residue pieces infested with *Fusarium* as a proportion of the total number of residue pieces analyzed.

Inoculum potential (IP) was calculated for each plot as the percentage of the residues infested with *Fusarium* spp. multiplied with the proportion (0–1) of the plot surface covered by residues after sowing. The percentage of the plot surface for the four treatments covered with residue after sowing are presented in **Table 1**, and also in Seehusen (2014).

**Table 1:** The average percentage of soil area covered with residues of the previous years’ oat crop, measured after sowing in spring and following the implementation of combinations of two residue treatments and four tillage treatments (Seehusen, 2014).

Location	Year	Straw removed after harvest				Straw chopped and retained in field			
		DAP^∗^	SSP	SAH	SSH	DAP	SSP	SAH	SSH
Solør	2011	2	3	7	26	4	3	11	49
Solør	2012	0	1	8	16	1	1	10	23
Østfold	2011	2	3	23	28	1	5	38	45
Østfold	2012	0	0	10	6	0	2	20	9

*^∗^Tillage regimes: DAP, deep autumn plowing; SSP, shallow spring plowing; SAH, shallow autumn harrowing; SSH, shallow spring harrowing.*

### Assessment of *Fusarium* DNA in Straw Residues

Surprisingly low percentages of *F. langsethiae*-infested residues (average field levels 0–1%) were recorded in our morphological analyzes of the *Fusarium* spp. associated with the oat residues examined, despite this species being commonly detected in Norwegian oat grains (Hofgaard et al., 2016). Therefore, the content of *Fusarium* DNA was quantified in the samples remaining after much of each sample was utilized for the morphological analysis.

The plant residues were milled using a ZM 200 Mill with a 0.2 mm sieve (Retsch, Haan, Germany). Total genomic DNA from one gram of milled residues was extracted using PowerMax^®^ Soil (MO Bio Laboratories, Inc., Carlsbad CA, USA) according to the manufacturers’ description. DNA was eluted in a volume of 5 ml and up-concentrated as follows: NaCl was added to a final concentration of 0.2 M, and the tube inverted 3–5 times to mix. Twenty microliters of linear acrylamide (5 mg/ml) was added, followed by the addition of 2.5 × the mix volume of cold 100% ethanol. The mix was inverted by 3–5 times and stored on ice overnight. The following day the mix was spun at 4500 × *g* for 35 min to pellet the DNA. The pellet was washed with 5 ml 70% ethanol and centrifuged 4500 × *g* for 10 min to re-pellet. The ethanol was decanted and the tube was inverted to drain, then turned right side up and air dried. The pellet was re-suspended in 200 μl of Solution C6 (MO Bio Laboratories), and cleaned using NucleoSpin^®^ Gel and PCR Clean-up (Macherey-Nagel, Dueren, Germany). DNA was eluted twice, in 30 μl each time, combined (total volume of 60 μl) and stored at -20°C prior to analysis by quantitative PCR (qPCR). Fungal standard DNA was extracted from pure cultures according to Divon et al. (2012) from the following isolates from the NIBIO collection: *F. avenaceum* (isolate ID 201081), *F. culmorum* (ID 201064), *F. graminearum* (ID 200630), and *F. langsethiae* (ID 201087).

The genomic DNA from plant residues were analyzed by TaqMan qPCR to determine the content of *F. avenaceum*, *F. culmorum*, *F. graminearum*, and *F. langsethiae* DNA, using the primers and probes described in Halstensen et al. (2006; *F. avenaceum*), Waalwijk et al. (2004; *F. culmorum* and *F. graminearum*), and Hofgaard et al. (2016; *F. langsethiae*). qPCR was performed in a total volume of 25 μl that consisted of 4 μl 10-fold diluted genomic DNA, 300 nM of each primer (Invitrogen by Thermo Fisher Scientific, Waltham, MA, USA), 100 nM of each probe, and 1 × SsoAdvanced^TM^ Universal Probes Supermix (Bio-Rad, Hercules, CA, USA), in a C1000 Touch Term Cycler combined with a CFX96TM Real-Time System (Bio-Rad). The probes for detection were labeled with 6-FAM (Applied Biosystems, by Thermo Fisher Scientific, Waltham, MA, USA) in case of *F. graminearum*, *F. langsethiae*, and *F. culmorum*, or Cy5 (Sigma–Aldrich, St. Louis, MO, USA) in case of *F. avenaceum*. All reactions were performed with the following parameters: 95°C for 3 min followed by 45 cycles of 95°C for 10 s and 60°C for 30 s. The data were analyzed using the Bio-Rad CFX manager software version 3.1 (Bio-Rad). In case of *F. langsethiae*, qPCR was performed on undiluted genomic DNA in addition to the 10-fold diluted DNA. The amount of fungal DNA in the samples was quantified as a mean of two technical qPCR replicates differing by a *C*q ≤ 1, using a standard curve algorithm with five dilutions of known amounts of DNA of the respective *Fusarium* species in the range of 0.001–4 ng. The fungal content is presented as pg fungal DNA per g plant residue (pg/g).

### Assessment of *Fusarium* DNA in Air Samples

Two Automatic Multi-Vial Cyclone Samplers (Burkard Manufacturing Co. Ltd., Rickmansworth, UK) were placed in each oat field in 2011 and 2012. One sampler was placed in plots with shallow harrowing in spring (SSH) where the straw was chopped, the other in deep autumn plowing (DAP) plots where the straw had been removed. The air intake of the spore traps was at about 1 m height above ground. Bio-aerosols were collected in 1.5 ml tubes at an air movement rate of 16.5 l/min, and the tubes were automatically replaced every 24 h (at 00.00 h). At Solør, air samples were collected from week 25 to 33 (the oat crop reached heading in week 28) in 2011, and from week 23 to 37 (heading: week 31) in 2012. At Østfold, samples were collected from week 19 to 33 (heading: week 26) in 2011, and from week 21 to 35 (heading: week 27) in 2012. The tubes were collected from the samplers once a week and stored at -20°C prior to DNA extraction.

Total genomic DNA was extracted from air samples collected over 1 week periods (equivalent to eight 1.5 ml tubes as the tubes were sampling only ½ day in the beginning and end of the period) using the FastDNA^®^ SPIN Kit for Soil (MP Biomedicals, Santa Ana, CA, USA). A volume of 489 μl of sodium phosphate buffer was successively transferred between all tubes included in one extraction, combined with vigorous vortexing of the buffer in the individual tubes, before transferring it to a Lysing Matrix E tube. The procedure was repeated once using the same amount of buffer, followed by a quick spin down of the tubes so that any remaining amounts of sodium phosphate buffer was also collected. Both buffer wash-troughs were combined into one extraction. DNA was extracted according to the manufacturer’s protocol, and eluted in 100 μl DNase/pyrogen-free water. Samples were stored at -20°C prior to analysis by qPCR.

Conidia from the standard isolates of *F. avenaceum*, *F. graminearum*, and *F. langsethiae* (see isolate ID’s above) were produced on mung bean agar medium (Dill-Macky, 2003) at 22–23°C under a combination of white and black light with 12 h photoperiod, and harvested after 10–15 days growth. The conidia were suspended in sterile water, and the spore concentrations were measured using KOVA^®^ Glasstic^®^ Slide 10 with Grids (Hycor Biomedical Inc.). Spore suspensions were stored at -20°C. For DNA extraction, spores suspensions were thawed on ice and 3–5 ml of spore suspension was centrifuged for 15 min at 3220 × *g*. Most of the water was removed without disturbing the spore pellet, and DNA was extracted using the FastDNA^®^ SPIN Kit for Soil (MP Biomedicals) as described above. The final concentration of these DNA standards was measured using a Qubit^®^ dsDNA HS Assay (Invitrogen) and a Qubit^®^ 2.0 Fluorometer (Invitrogen). The DNA standards were stored at -20°C prior to use in qPCR.

*Fusarium avenaceum*, *F. graminearum*, and *F. langsethiae* DNA was quantified by qPCR as described above, except that in this case the analysis was performed on undiluted DNA extracts from air samples. The amount of fungal DNA in the air samples was quantified using a standard curve algorithm of five dilutions of conidial DNA from the respective species’ standard. The dilutions were in the range of 0.1–400 pg *F. graminearum* DNA (equivalent to 0.6–2,565 conidia), and 0.2–800 pg of both *F. avenaceum* and *F. langsethiae* DNA (equivalent to 10–40,000 conidia for each species). The amount of fungal DNA was expressed as pg *Fusarium* DNA per week.

Little *Fusarium* DNA was detected in the spore traps prior to heading. In case this may have been a result of the spore-traps’ inability to catch spores released from plant residues near the soil surface, only data from *Fusarium* DNA recorded from the week of heading onward were used in the subsequent analyses. The sampling in each field was terminated from 1 to 6 weeks prior to harvest.

### Weather Data

For both field locations, weather data were collected from the nearest Agrometeorology Norway^1^ weather station. Historical values, covering 1961 to 1990, for the weeks sampled, were provided by the Norwegian Meteorological Institute (Supplementary Table S1).

In order to identify possible associations between weather conditions and the weekly amounts of *Fusarium* DNA collected in spore traps, weather data were collected at each location from 2 weeks prior to cereal heading till the end of the spore trapping period. Hourly recorded data on temperature (°C), precipitation (mm), relative humidity (%) and wind speed (m/s), were summarized into different weather variables using MATLAB (2013b). Daily precipitation and temperatures in the period from 2 weeks prior to cereal heading until end of spore trapping are presented in **Table 2**.

**Table 2:** Minimum, maximum and mean daily precipitation (mm) and temperature (°C) in the period from 2 weeks prior to cereal heading until end of spore trapping at two locations (Solør and Østfold) for 2 years (2011 and 2012).

Location	Year	Weeks	Daily precipitation, mm			Temperature, °C		
			Min	Max	Mean	Min	Max	Mean
Solør	2011	26–33	0	31.80	3.8	9.5	20.2	15.8
Solør	2012	29–37	0	24.6	3.3	7.4	19.2	13.7
Østfold	2011	24–33	0	55.8	3.9	11.4	21.3	16.1
Østfold	2012	25–35	0	32.0	3.2	11.3	19.0	14.5

### Statistical Analysis

The percentage infestation of *F. avenaceum* and *F. graminearum* on residue and the calculated IP per plot within each field experiment were subjected to statistical analysis. No analyses were performed for the IP of *F. culmorum* or *F. langsethiae* due to the low levels recorded in straw residues. Data from 2011 and 2012 were analyzed separately. Tillage treatment (nested within whole plots) and straw removal (whole plots, nested within blocks) were used as factors in the statistical model in which block was used as a random factor. Significant treatment effects were separated by applying proc mixed in SAS program for Windows (version 9.3, SAS institute inc.) creating pairwise comparisons and 95% confidence intervals according to Tukey–Kramer’s method.

Regression analysis (R 3.2.0) were performed in order to identify possible associations between weather conditions and the weekly amount of *F. avenaceum* and *F. graminearum* DNA collected in spore traps. No analysis was performed for *F. langsethiae* due to the low DNA levels recorded. Weather data for air temperature (°C), precipitation (mm) and relative humidity (%) was collected on an hourly basis but the regression conducted on blocks of data combined in three formats: from the week of spore collection, from the week prior to the start of the spore collection period, and from the 2 weeks prior to the start of the spore collection period. The average amount of *Fusarium* DNA detected in the two spore traps sampling within a single field during a 1 week period was used in the analysis.

Regression analysis (Minitab 16) were performed in order to identify possible associations between the percentage of oat straw residues within a field plot infested with *F. avenaceum* versus *F. graminearum*, and the amount of *F. avenaceum* DNA vs. *F. graminearum* DNA per gram straw residues within a field plot. As well to identify possible associations between the DNA content of *F. avenaceum, F. graminearum, F. culmorum*, or *F. langsethiae* per gram straw (determined by qPCR), and the percentage of the straw infested by these fungal species, respectively, within the same field plot. In the regression analysis, the natural logarithm (ln) of *Fusarium* DNA (pg DNA per gram plant residue) was used. qPCR values of *F. langsethiae* and *F. culmorum* were added a value of 1 prior to ln transformation due to low DNA levels.

## Results

### Assessment of Straw Residues

The percentage of the soil area covered by oat straw residues differed between residue treatment and tillage regimes (**Table 1**). At both locations, a higher amount of straw was recorded in 2011 compared to 2012. In 2011, the average percentage of residue cover at Solør ranged from 2 to 49% and at Østfold from 1 to 45%, depending on the residue treatment and tillage regimes. In 2012, the average percentage of residue cover ranged from 0 to 23% at Solør, and from 0 to 20% at Østfold.

### *Fusarium* Isolated from Residues

**FIGURE 1:**
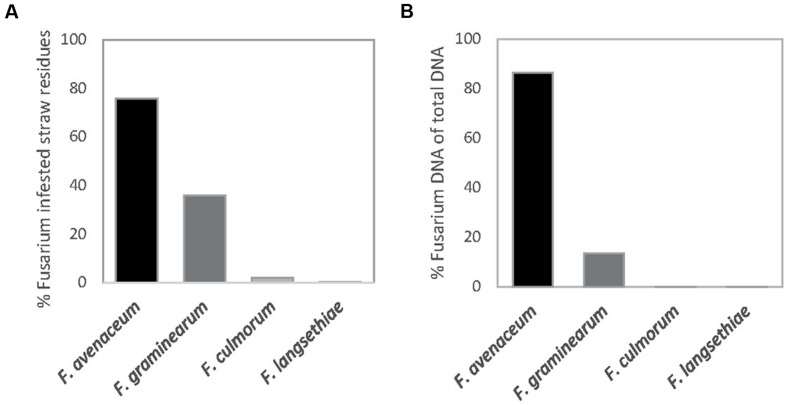
**Percentage of oat straw residues from the previous years’ crop from which *Fusarium* was isolated (A).** The relative amount of DNA of different *Fusarium* species given in percentage of total *Fusarium* DNA quantified in these residues **(B).** The straw residues were collected from the soil surface at sowing at Solør and Østfold in 2011 and 2012.

**FIGURE 2:**
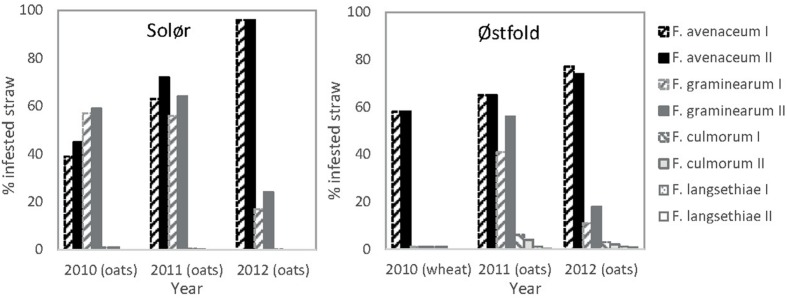
**Percentage of straw residues of oats or wheat from the previous years’ crop from which *Fusarium* was isolated.** The residues were collected on the soil surface at sowing at Solør and Østfold in the years 2010, 2011, and 2012. The previous crop is indicated in brackets. Hatched bars represent data from plots where most of the straw was removed in autumn (I). Filled bars indicate that the straw was chopped and retained in the field in autumn (II). The gray tones of the bars relates to the different *Fusarium* species.

**FIGURE 3:**
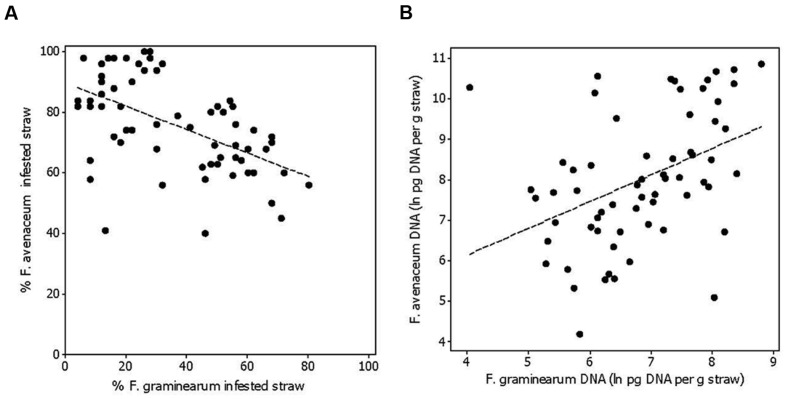
**The relationship between the percentage of oat straw residues within a field plot infested with *Fusarium avenaceum* and *F. graminearum* (*R*^2^_adj_ = 27%, *p* < 0.001) (A), and the relationship between the amount of *F. avenaceum* DNA compared to *F. graminearum* DNA calculated as ln pg DNA per gram of straw residue (*R*^2^_adj_ = 16%, *p* = 0.001; B).** The residues were collected after sowing in spring at Solør and Østfold locations in the years 2011 and 2012.

*Fusarium avenaceum* was the most prevalent *Fusarium* species isolated from straw residues at both field locations over the 3-year period (**Figures 1** and **2**). For both fields, the yearly average *F. avenaceum* infestation of residues increased during the experimental period (**Figure 2**). At Solør the percentage of straw residues infested with *F. avenaceum* increased from 42 to 94% during the experiment, and at Østfold, an increase from 58 to 75% was observed (**Figure 2**). The proportion of *F. avenaceum* infested straw residues ranged from 40 to 100% between the different treatments (median 76%) in 2011 and 2012 (**Figure 3A**).

*Fusarium graminearum* was the second most common *Fusarium* species isolated in this study (**Figure 1A**). The percentage of *F. graminearum* infestation on straw residues in spring declined from 2011 to 2012 at each location (**Figure 2**). The proportion of oat straw infested with *F. graminearum* ranged from 4 to 80% between the different treatments (median of 31%) across 2011 and 2012 (**Figure 3A**). In some location-years, a slightly lower *Fusarium* infestation of straw was observed on average for treatments where the straw was removed compared to those where straw was retained (**Figure 2**). This effect was most evident in the case of *F. graminearum*. However, the percentage of straw within a field infested by all *Fusarium* spp. was not significantly influenced by tillage or residue management treatments. The percentage of *F. avenaceum* infested straw was negatively correlated with the percentage of *F. graminearum* infested straw (*R*^2^_adj_ = 27%, *p* < 0.001, **Figure 3A**).

Only low levels (less than 5%) of *F. culmorum* were detected on the straw residues at both locations (**Figure 2**). *F. langsethiae* was only detected in Østfold (2011 and 2012) and then only at average levels below 1% (**Figure 2**). *F. tricinctum, F. cerealis, F. sporotrichioides, F. poae*, and *F. equiseti* were also detected, but their average levels generally fell below 2% (data not shown). One exception was Østfold in 2012, where *F. tricinctum* was isolated from 5% of the oat straw residues sampled.

### *Fusarium* DNA Quantified in Residues

**FIGURE 4:**
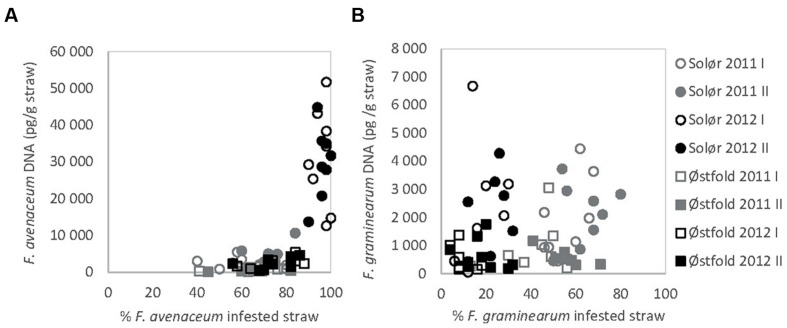
**Association between the DNA content of *F. avenaceum* (A) and *F. graminearum* (B) per gram straw (pg DNA/g straw), analyzed by qPCR, and the percentage of *Fusarium*-infested straw within the same field plot, determined by morphological analyzes**.

The DNA of *F. avenaceum, F. graminearum, F. culmorum*, and *F. langsethiae* was quantified in straw residues by using qPCR (**Figure 1B**). In general, the highest levels of DNA were detected for *F. avenaceum* followed by *F. graminearum*. DNA of *F. culmorum* and *F. langsethiae* was detected at low levels, and accounted on average for less than 1% of the total DNA quantified for these four *Fusarium* species. DNA of *F. langsethiae* was sporadically detected in all fields, but always at low levels (0–2.2 pg per gram straw residue within plot). DNA of *F. culmorum* was detected in all fields, and at variable levels (0–144 pg per gram straw residue within plot). For most samples, the DNA content of a particular *Fusarium* species was below 10,000 pg per gram straw residue (**Figures 4A,B**). One exception was the field at Solør in 2012, where the DNA content of *F. avenaceum* ranged from 12,620 to 51,695 pg per gram straw residue (**Figure 4A**). By comparison, DNA levels of *F. langsethiae* never exceeded 2.2 pg per gram straw residue (data not shown). A positive relationship was calculated between the quantities of DNA detected for *F. avenaceum* and *F. graminearum* calculated as ln pg *Fusarium* DNA per g plant residue (*R*^2^_adj_ = 16%, *p* = 0.001, **Figure 3B**). For samples in which less than 90% of the straw residues were infested with a specific *Fusarium* species, the DNA content per gram straw ranged from 0 to 10,000 pg per gram straw (**Figures 4A,B**). For samples where more than 90% of the residues were infested with *F. avenaceum*, concentrations of *F. avenaceum* DNA were consistently above 10,000 pg/g (**Figure 4A**). A significant, positive association (*R*^2^_adj_ = 54%, *p* < 0.001) was found between the DNA content for *F. avenaceum* (ln pg DNA per g plant residue) and the percentage of residues infested by *F. avenaceum* within the same field plot. A low but significant positive association (*R*^2^_adj_ = 24%, *p* < 0.001) was also found between the DNA content for *F. culmorum* ln (pg DNA per g plant residue + 1) and the percentage of residues infested by *F. culmorum* within the same field plot. No significant associations were found between the DNA content for *F. graminearum* and *F. langsethiae* and the percentage of residues infested by these *Fusarium* species within the same field plot.

### Inoculum Potential of *Fusarium* in Relation to Tillage and Residue Management

Tillage significantly influenced the IP of both *F. avenaceum* and *F. graminearum* at most locations, whereas no significant effects of straw removal were evident. However, significant interactions between straw removal and tillage regime were found within many fields. Therefore, a Tukey analysis was performed to identify significant differences in IP of *F. avenaceum* and *F. graminearum* between combined treatments (straw removal combined with tillage treatment).

**FIGURE 5:**
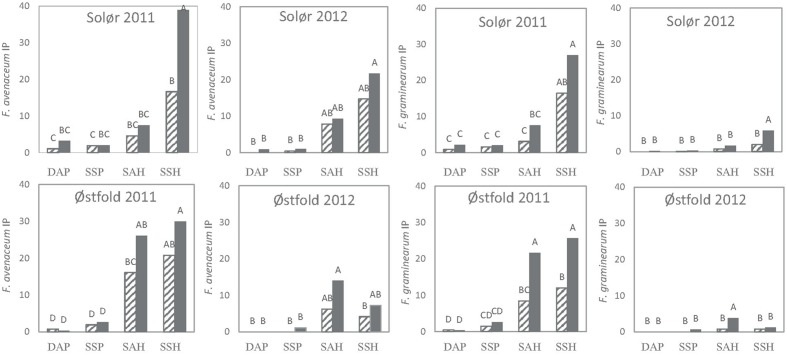
**Effects of tillage treatment and residue regimes on the inoculum potential (IP) of *F. graminearum* and *F. avenaceum* in two experimental fields of oats in southeast Norway (Solør and Østfold) in 2011 and 2012.** Tillage treatments include DAP, deep autumn plowing; SSP, shallow spring plowing; SAH, shallow autumn harrowing; SSH, shallow spring harrowing. Data from the two residue regimes examined are indicated by either hatched bars, where the straw was removed, or filled bars, where the straw was chopped and retained in the field. IP was calculated as the relative soil area covered with oat residues after sowing in spring (0–1) multiplied by the percentage of the residues infested with the respective *Fusarium* species. Different letters indicate significant treatments effects at *p* = 0.05.

Generally, higher IPs were estimated for harrowed compared to plowed plots (**Figure 5**). Within individual locations, the highest IPs were found on spring-harrowed plots where the straw was retained after harvest. In fields where the IPs exceeded 10% in spring-harrowed plots (mostly recorded in 2011, **Figure 5**), significant differences in IP were often recorded between harrowed and plowed plots. Moreover, in some of these spring-harrowed plots, straw removal significantly reduced the IP of *F. avenaceum* (Solør, 2011) and *F. graminearum* (Østfold, 2011; **Figure 5**). Hardly any significant effect of tillage on the IP was detected in fields where the *Fusarium* spp. were isolated from less than 25% of the residues sampled, as was the case for *F. graminearum* in Østfold and Solør in 2012 (**Figures 2** and **5**). Similarly, hardly any significant effect of soil cultivation was detected when the different tillage treatments resulted in a maximum plant residue cover below 20% as Østfold 2012 (**Table 1** and **Figure 5**). The lowest IPs (<5%) were estimated for plowed plots where the removal of straw residue or timing of tillage treatment had no apparent impact upon the *Fusarium* IP. No significant differences in IPs were found between spring and autumn plowed plots.

### *Fusarium in* Air Samples

**FIGURE 6:**
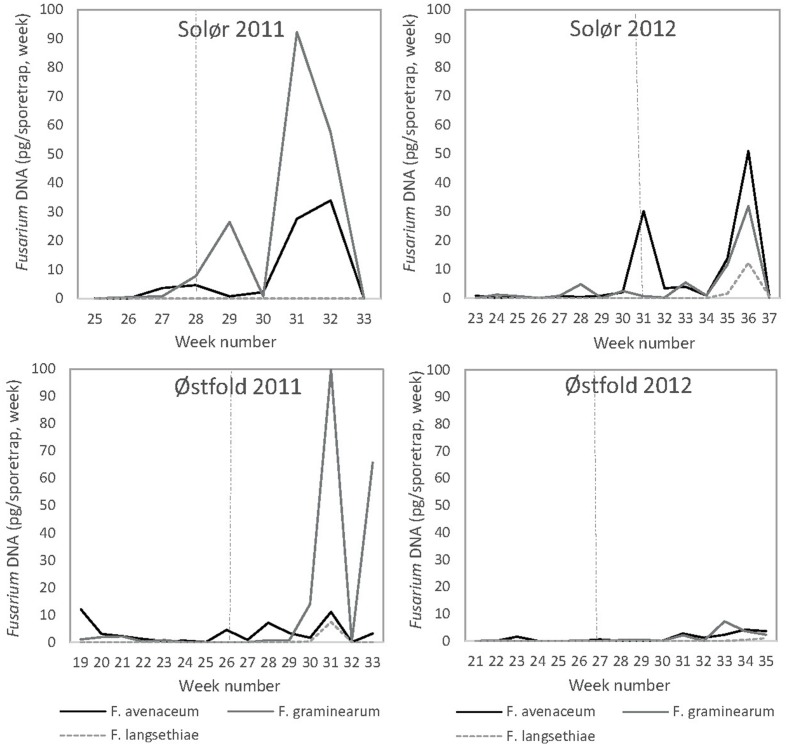
**The weekly average DNA amounts (pg) of *F. avenaceum, F. graminearum*, and *F. langsethiae* recorded in spore-traps located in oat trials at two locations in southeast Norway (Solør and Østfold) in 2011 and 2012.** *Fusarium* DNA was quantified by qPCR. Stippled lines indicate the timing (given as the week) of oat heading.

As no consistent differences were detected in the amount of *Fusarium* DNA collected in the two spore traps situated in plots with different tillage treatments within a field, average values of fungal DNA collected per week are presented for each field. *F. langsethiae* DNA was only detected at low levels, with an average maximum level of 12 pg DNA in week number 36 at Solør 2012 (**Figure 6**). *F. langsethiae* DNA was only recorded in the spore traps at late time points, from 4 weeks after heading. DNA of *F. avenaceum* and *F. graminearum* was detected at higher levels than that of *F. langsethiae*. At both locations, the amount of *F. graminearum* DNA was, however, less in 2012 than in 2011 and only low levels of *F. graminearum* DNA were detected at Østfold in 2012. For *F. avenaceum*, the highest DNA levels were detected at Solør, with an average maximum of 51 pg in week 36, 5 weeks after heading, in 2012. Only low levels were detected at Østfold in both years. The average amount of *F. avenaceum* and *F. graminearum* DNA recorded increased significantly in the sampling periods after heading in spore-traps at both locations in 2011 and 2012 (**Figure 6**). The highest *F. graminearum* DNA amounts were recorded in 2011, with an average maximum of 100 pg DNA in week number 31 in the two spore traps located at Østfold (**Figure 6**).

### *Fusarium* Dispersal in Relation to Weather Conditions

Wetness and moisture in the period prior to spore sampling influenced the amount of *F. avenaceum* DNA in spore traps sampling from heading onward. The best regression model to explain the variations in *F. avenaceum* DNA comprised the number of days with precipitation in the two consecutive weeks prior to spore sampling, the mean daily hours with relative humidity exceeding 70% during the week of spore sampling and their interaction. This model accounted for 53% of the variation in the data, with an *R*^2^_adj_ = 47%. Both main terms in the model were positive, giving a positive relationship for both factors separately. However, only the precipitation term was significant (*p* < 0.05). The interaction term was negative (*p* < 0.05), indicating that the effects of these factors were not additive.

Lower associations were generally found between weather conditions and the DNA of *F. graminearum* collected. The best model to explain the variation in *F. graminearum* DNA included data on the total precipitation during the two consecutive weeks prior to spore sampling, the mean air temperature during the week of spore sampling and the mean air temperature 1 week before spore sampling, in addition to the four interaction terms. The model described 59% of the variation in the data, with an *R*^2^_adj_ = 46%. All the main factors in the model were positive, but only the precipitation factor was significant (*p* < 0.05). The three factor interaction was positive and significant (*p* < 0.05), while all the two factor interactions were negative and only significant when precipitation was included in the interaction. The relative amount of airborne *Fusarium* inoculum increased after rainy periods and corresponded, in most cases, with the IP calculated within a field in spring.

## Discussion

The objective of this work was to elucidate the influence of different tillage and straw coverage regimes on the IP and dispersal of *Fusarium* spp. in Norwegian spring oats. In general, the amount of crop residues left on the soil surface and thus, the IP, was significantly lower on plowed compared to harrowed plots. On harrowed plots, the removal of straw prior to harrowing reduced the IP. Differences in the relative *Fusarium* spp. infestation of oat straw were detected between locations and years. The relative amount of airborne *Fusarium* inoculum increased after rainy periods and corresponded in most cases with the IP calculated within a field in spring. *F. langsethiae* was only detected sporadically and at low levels in residues (DNA and fungal growth) and spore traps (DNA).

Soil cultivation clearly influenced the *Fusarium* IP. For both *F. avenaceum* and *F. graminearum*, the lowest IPs were found on the plowed plots and the highest IPs on unplowed, spring harrowed plots. This is in agreement with other studies that, based on analysis of crop stubble, reported reduced inoculum levels of *F. graminearum* in plowed fields /experimental plots compared to those established with reduced or minimum tillage (Dill-Macky and Jones, 2000; Guo et al., 2010). In our study, the overall *Fusarium* IP was most closely related to the level of *Fusarium* infested straw within a field, with no large differences in the percentage of *Fusarium* infested straw recorded between plots subjected to different tillage treatments within a field. The observed differences in *Fusarium* IP between treatments within a field were more closely related to the amount of straw residues on the soil surface. This finding is supported by Maiorano et al. (2008), who observed that the amount of *Fusarium* spp. in harvested wheat grain mainly corresponded to the amount of residues lying on the soil surface of the production field, rather than the tillage regime. In our study, significant effects of tillage treatments on IPs were largely recorded in 2011. The amount of straw residues left on the soil surface after tillage operations in the spring of 2011 was greater than in spring 2012, which likely explains the greater difference observed in 2011. Similarly Koch et al. (2006) reported only slight differences in *Fusarium* disease development between tillage treatments when residue cover was below 30%. In our study, significantly lower IPs were often recorded for autumn harrowed plots compared to spring harrowed plots within the same field, although the time of plowing (autumn vs. spring) did not significantly influence the IP of the plowed treatments. The observed differences thus largely reflect the variation in straw cover resulting from these various tillage methods (Seehusen, 2014).

Removal of straw residues generally reduced the IP in harrowed treatments, though the effect was not always significant. By contrast, the removal of straw residues did not significantly influence the IP on the plowed treatments. The reason for the different effect of straw removal in harrowed vs. plowed treatments is most probably the generally low amount of residues, and the correspondingly low IPs, in the plowed treatments. Our results are in line with the general understanding of plowing as the best means of reducing the occurrence, and thereby the IP, of *Fusarium* spp. in cereal fields (Dill-Macky and Jones, 2000; Champeil et al., 2004b; Guo et al., 2010). The effect of tillage on FHB development may not always be evident, as weather factors play an important role in the dispersal and infection of *Fusarium* spp. (Lori et al., 2009; Prussin et al., 2014a). According to our data, spring plowing appears to be the best option to both reduce the risk of soil erosion and at the same time minimize *Fusarium* diseases. Removal of cereal straw in autumn would be the best practice for reducing the IP of *Fusarium* spp. where harrowing is the preferred tillage system.

In our study*, F. avenaceum* was the dominant *Fusarium* species recovered from oat residues, followed by *F. graminearum*. In contrast, *F. culmorum* was detected in only a few of the residues sampled. These *Fusarium* species have been recorded on wheat residues elsewhere (Köhl et al., 2007; Golkari et al., 2008; Landschoot et al., 2011; Postic et al., 2012), as well as on oat residues in Canada (Golkari et al., 2008). High levels of *F. sporotrichioides* were also detected on oat residues in the Canadian study. The discrepancy between the Norwegian and Canadian studies may be explained by the generally low levels of *F. sporotrichioides* normally detected in Norwegian cereals (Bernhoft et al., 2010). *F. langsethiae* was detected in our field trials, but at exceedingly low levels despite the fact that this fungal species and the mycotoxins it produces are commonly detected in oats grown in Norway (Aamot et al., 2013; Hofgaard et al., 2016). The *Fusarium* species detected on the oat residues in this study reflected those most commonly detected in Norwegian grain, other than *F. langsethiae* (Kosiak et al., 2003; Henriksen and Elen, 2005; Bernhoft et al., 2010; Norwegian Scientific Committee for Food Safety, 2013; Hofgaard et al., 2016). This supports previous findings that have identified crop residues as an important source of inoculum for FHB (Dill-Macky and Jones, 2000).

The general increase in *F. avenaceum* infestation of crop residues in our fields during the project period is in agreement with another report of increased *F. avenaceum* infestation of crop residues in monoculture cereals (Fernandez et al., 2008). *F. avenaceum* infestation of wheat residues is reported to be more stable over time than that of *F. graminearum* (Köhl et al., 2007; Hogg et al., 2010; Palazzini et al., 2013). Similarly while we observed a yearly increase in the relative prevalence of *F. avenaceum*, we did not see *F. graminearum* increase on straw residues over the period of this study. Infestation of crop residues by plant pathogens may be related to the establishment of fungi in host tissues prior to crop senescence (Bruehl and Lai, 1966; Köhl et al., 2007; Hogg et al., 2010). The low *F. graminearum* infestation of residues in 2012 does not appear to be explained by a lack of inoculum the previous year, as the DNA levels in spore traps were high, but the lower infestation of residues by *F. graminearum* may be explained by the relatively late peak spore dispersal, compared to the cereal flowering period, that would likely have resulted in relatively few FHB infections.

*Fusarium avenaceum*, had less marked peaks of spore dispersal than *F. graminearum* in 2011, but there was a relative stable dispersal of spores from shortly before heading till the end of the spore sampling period. Since we were not able to differentiate between spore types (ascospores vs. conidia), we elected to use the amount of DNA (in pg), as an indication of the relative abundance of the different species. The use of DNA as a measure makes it difficult to extrapolate the relative number of spores of the two species as the DNA level in spores may vary with species and spore type. Our result do, however, suggest that *F. avenaceum* produced inoculum more consistently and over a longer period, than *F. graminearum*, in 2011.

The differences we detected in the relative prevalence of *Fusarium* species on crop residues in spring, may be related to the establishment of the fungi as pathogens in the living plant. The infection of above ground plant tissues by *Fusarium*, and thus the infestation of crop residues detected later, is likely dependent upon the prevailing weather conditions from flowering until harvest (McMullen et al., 2012). The monthly average temperature during cereal flowering (July) in our field trials in 2010–2012, ranged from 14.8 to 17.4°C. This is closer to the optimal growth temperature for *F. avenaceum* (20°C) compared to *F. graminearum* (25°C; Brennan et al., 2003). Although *F. graminearum* has a higher *in vitro* growth rate compared to *F. avenaceum* in the range 10–30°C, the growth rate of *F. avenaceum* is reported to be less affected by temperature than that of *F. graminearum* (Brennan et al., 2003). Consequently, in addition having temperatures closer to the optimum for growth of *F. avenaceum*, small fluctuations in the temperature likely influenced the growth and establishment *F. avenaceum* less than *F. graminearum* in our trials.

The lower proportion of *F. graminearum* infested straw at all locations in 2012 compared to 2011 may also be explained by the influence of weather conditions between harvest and the sowing of the subsequent crop. The competition between *Fusarium* and other microorganisms in crop residues is influenced by environmental conditions, especially temperature and moisture (El-Naggar et al., 2003; Lakhesar et al., 2010; Leplat et al., 2013). A sharp reduction in *Fusarium* infestation on cereal residues has been observed in spring, and survival of *F. graminearum* seems inversely related to the decomposition of residues (Pereyra et al., 2004; Köhl et al., 2007). The recovery and sporulation of plant pathogens from residues have been reported to decrease in warm and wet conditions, probably due to an increased decomposition rate combined with increased antagonistic activity (Zhang and Pfender, 1992; Lakhesar et al., 2010). In our study, the weather at Solør and Østfold in September 2011 was warmer (11.6 and 12.7°C, respectively) and wetter (119 and 236 mm, respectively) compared with the previous year with average temperatures of 9.6C and 10.9°C and rainfall of 44 and 99 mm, respectively. In addition, the precipitation before sowing in the spring (April) at Solør and Østfold was higher in 2012 (28 and 87 mm, respectively) than in 2011 (15 and 45 mm, respectively) and this may also have increased the activity of antagonistic microorganisms. We surmise that the relatively warm and moist conditions during autumn 2011 along with the moist conditions in spring 2012 may have facilitated both the decomposition of plant residues and competition by other saprophytes that reduced the survival of *F. graminearum* in those residues.

Although not significant, we observed a consistent trend of a higher percentage of *F. graminearum* infested oat straw residues on plots where the straw was retained after harvest than on plots where most of the straw was removed. This may be explained by a relatively lower proportion of the residues in direct contact with the soil when the straw is retained after harvest as *F. graminearum* survives and reproduces better on surface residue than on buried residue (Khonga and Sutton, 1988; Pereyra et al., 2004). A comparison of *F. graminearum* infested residues in autumn vs. spring would have been of interest.

We found a negative association between the infestation of residues by *F. avenaceum* and *F. graminearum*. This finding is similar with the relative abundance reported for these two species in Norwegian grain determined using morphological methods (Kosiak et al., 2003; Bernhoft et al., 2010). The negative association may be indicative of competition between these two fungal species. *F. graminearum* has a faster growth rate than *F. avenaceum* at the temperatures used during the incubation in our study (Brennan et al., 2003), which may explain why we detected a reduced *F. avenaceum* infestation in residues that were highly infested with *F. graminearum*. This explanation is supported by the qPCR analysis, where a low but significant positive association was detected between the DNA content of *F. avenaceum* vs. *F. graminearum* in the residues examined. This suggests that the observed data on infestation of cereals or plant residues by different *Fusarium* species may be influenced by growth rates of the individual *Fusarium* species in the sample.

Despite the use of both morphological and molecular methods, little *F. langsethiae* was detected in the residues collected in our study. Furthermore, we could not find any association between the IP of *F. langsethiae* in crop residues and the amount of fungal DNA detected in the spore traps. The epidemiology of *F. langsethiae* is unclear (Imathiu et al., 2013a), and our study adds little to our understanding of the sources of inoculum of *F. langsethiae*.

The relative amounts of total *Fusarium* spp. isolated from the residues in this study corresponded with the total DNA quantified of the *Fusarium* species in residues. A significant association was found between the DNA content of *F. avenaceum*, and the percentage of straw infested by *F. avenaceum.* DNA content of *F. culmorum* was slightly associated with the percentage of straw infested by *F. culmorum.* However, no associations were found between the DNA content of *F. graminearum* or *F. langsethiae* and the percentage of straw infested by these fungal species. This poor correlation may be partly due to variation within the sample as the *Fusarium* DNA was quantified from the residues remaining after straw was selected from the sampled straw for isolation and morphological analysis. The qPCR was initiated to investigate if *F. langsethiae*, detected at extremely low levels in our isolations was being out-competed by the faster growing *Fusarium* species. Additionally the qPCR enabled finer scale quantification of *Fusarium* species, whereas in the isolations the percentage of straw infested with a specific *Fusarium* was limited by the number of residue pieces examined. On the contrary, qPCR quantified the amount of fungal DNA irrespective of how this DNA was distributed in a sample or whether the fungus was dead or alive. qPCR has been used by others to record the change in the relative abundance of *Fusarium* species in plant residues over time (Köhl et al., 2007; Hogg et al., 2010). However, it is recognized that obtaining amplifiable DNA from crop residues may be challenging (Imathiu et al., 2013b). We suggest that additional studies need to be undertaken to investigate whether molecular methods such as qPCR are appropriate to quantify the IP of *Fusarium* in cereal residues.

According to our regression models, the DNA and thus the airborne spores of *F. avenaceum* and *F. graminearum* increased after rain periods. The amount of *F. avenaceum* DNA collected in the spore traps increased with the number of rain days in the previous 2 weeks, and with higher mean daily hours with RH > 70% during the week of spore sampling. Increases in the amount of airborne *F. graminearum* spores was, by contrast, related more closely to the total precipitation in the 2 weeks prior to spore sampling. Although the variables that influenced spore dispersal varied, an increase in the DNA sampled of both these fungal species was related to an increase of humid/wet conditions, which corresponds well with other studies (de Luna et al., 2002; Xu et al., 2008; Manstretta and Rossi, 2015). For *F. graminearum*, model performance also increased when data for mean air temperature the week before and during spore sampling were included, whereas the strength of the *F. avenaceum* model did not increase when temperature data was included. This may indicate that growth and sporulation of *F. avenaceum* was less affected by temperature within the temperature range (9–21°C) of this study than was *F. graminearum*. *F. avenaceum* is known to have a lower optimum temperature for conidia production than *F. graminearum* (Rossi et al., 2002). However, since we did not examine the samples morphologically, we cannot know if conidia or ascospores were being collected in our study. Differences in temperature response was also reflected in reports of *in vitro* growth of these two fungal species (Brennan et al., 2003). Our findings are supported by studies demonstrating that *F. graminearum* perithecia number and development was enhanced by increasing temperatures between 12 and 20°C (Dufault et al., 2006). Our study indicate that the release of spores of *F. graminearum* increased with increasing rain combined with increasing temperatures up to 21°C. The higher levels of *F. graminearum* detected in 2011, may be explained by higher temperatures during the period of spore trapping in this year of the study.

The relative amount of airborne *Fusarium* inoculum recorded in a field in our study from before heading till the end of the spore trapping period, generally corresponded to the IP in the treatments that were harrowed in the spring. The *Fusarium* DNA was most abundant in the air samples collected after heading. As the air intake of the spore traps was at 1 m height above ground level, our spore traps may be unsuitable for capturing spores released from *Fusarium*-infested residues in the immediate vicinity. Inoculum generated from residues may be better captured by using sampling nearer to the residue (Munger et al., 2014; Prussin et al., 2014b). We detected no consistent differences in the amount of *Fusarium* DNA collected in the spore traps situated in plots with different tillage treatments, which may indicate that the data are more representative of the background inoculum in the field rather than the inoculum of individual treatments. Small and inconsistent differences in *F. graminearum* colony-forming units were also detected in Petri dish spore traps placed in plots with different tillage and cropping systems in a Canadian study (Munger et al., 2014). A comparison of *F. graminearum* and *F. avenaceum* DNA amounts from different types of spore-traps co-located would be of interest, as well as a more thorough study of the kind of spores (conidia or ascospores) collected by the different spore traps.

Disease development and the contamination of grain by *Fusarium* spp. and mycotoxins were not examined in our study, and therefore, we can only speculate on whether the differences in *Fusarium* IPs influenced these variables. However, other studies have described relationships between cropping practices, inoculum levels and development of FHB in monoculture cereals (Guo et al., 2010). An effect of tillage regimes was also reported to explain differences in *Fusarium* spp. isolated from cereal grains of barley and oats in a Norwegian study (Henriksen, 1999), which indicated that the IP within a field impacts the level of *Fusarium* spp. detected in grain harvested from that field. The effect of plowing on reducing the *Fusarium* inoculum within a field is frequently unclear (Miller et al., 1998; Munger et al., 2014). That *Fusarium* inoculum may be transported aerially over large distances is well recognized (Lori et al., 2009; Prussin et al., 2014a) suggesting that inoculum delivered by long-distance spore dispersal may overwhelm local sources of inoculum. Presumably, inoculum pressure over a large area can be reduced if sources of inoculum within a region are diminished through the cumulative effects of reducing inoculum at the individual field level.

## Conclusion

The *Fusarium* species most prevalent on oat residues in this study reflected those species most commonly detected in Norwegian oat grains although *F. langsethiae*, which is often isolated from Norwegian oats, was rarely detected. Our findings support previously published work that have identified crop residues, principally straw, as an important source of *Fusarium* inoculum. Our results are also in line with the general understanding of plowing as a means of reducing the IP of *Fusarium* spp. In areas where spring plowing is feasible, this appears to be the best option for Norwegian growers to reduce the risk of soil erosion while minimizing the risk of *Fusarium* infection. If harrowing is preferred, removal of the straw of a cereal crop prior to harrowing should aid in reducing the IP of *Fusarium* spp. This is the first report of *F. graminearum*, *F. avenaceum*, and *F. langsethiae* recorded in air sampled in cereal fields in Norway. The amount of airborne *Fusarium* increased after rainy periods, a finding which corresponds well with other studies. As airborne *Fusarium* detected at flowering generally corresponded with the IP of straw residues measured in the spring of our study, it appears that tillage practices aimed at reducing straw residues could be recommended to reduce the subsequent risk of *Fusarium* infection of cereal hosts and the subsequent contamination of grain by mycotoxins. Our results will be valuable in the development of agricultural decision support systems aimed at reducing the risk of *Fusarium* infection in cereals in Norway.

## Author Contributions

IH: Responsible for coordinating the work (planning, implementation, and interpretation). Responsible for writing this manuscript. TS: Responsible for coordinating the work connected the field trials. Responsible for recording the proportion of the soil surface area covered with straw residue. Involved in discussions and the writing of this manuscript. HA: Responsible for all DNA analysis. Involved in planning, discussions and in the writing of this manuscript. HR: Involved in the work connected to planning and implementation of the field trials, discussions and in the writing of this manuscript. JR: Responsible for all morphological analysis of *Fusarium*. Involved in planning, discussions and in the writing of this manuscript. VL: Responsible for the technical equipment and for coordinating the work connected to the collection of debris for *Fusarium* analysis and of bio-aerosols from spore traps. Involved in planning and discussions. A-GH: Responsible for analyzing the association between weather conditions and *Fusarium* inoculum collected in spore traps. Involved in discussions and in the writing of this manuscript. RD-M: Involved in the planning of this work, discussions, interpretation of the results, and in the writing of this manuscript. GB: Project leader. Involved in the planning of this work, in discussions regarding methods and implementation of the results, and in the writing of this manuscript.

## Conflict of Interest Statement

The authors declare that the research was conducted in the absence of any commercial or financial relationships that could be construed as a potential conflict of interest.

## Footnotes

1 http://lmt.nibio.no
