# Supplementary material for: Inoculum Potential of Fusarium spp. Relates to Tillage and Straw Management in Norwegian Fields of Spring Oats
Source: Front Microbiol. 2016 Apr 22;7:556. doi: 10.3389/fmicb.2016.00556 (PMC4841101; doi:10.3389/fmicb.2016.00556)
Supplement: Supplementary file 1 [file Table_1.DOCX]

**Supplementary info,**

**Table S1.** Monthly average temperature (temp.) and precipitation (prec.) and normal values (Ø) in the period 1961-1990 at three locations (Solør and Østfold) in southeast Norway during a four-year period (2009-2012).

| **Temp. (˚C)** | Solør | | | | | Østfold | | | | |  | | | | |
| --- | --- | --- | --- | --- | --- | --- | --- | --- | --- | --- | --- | --- | --- | --- | --- |
| Month | 2009 | 2010 | 2011 | 2012 | Ø | 2009 | 2010 | 2011 | 2012 | Ø |  |  |  |  |  |
| January | -4.1 | -13.0 | -7.3 | -5.7 | -7.7 | -1.7 | -9.2 | -4.3 | -1.9 | -3.8 |  |  |  |  |  |
| February | -8.5 | -9.8 | -7.1 | -4.9 | -7.1 | -5.6 | -7.1 | -5.5 | -2.6 | -3.7 |  |  |  |  |  |
| March | 0.4 | -2.0 | -0.8 | 3.4 | -2.0 | 0.3 | -2.0 | 0.3 | 4.4 | -0.2 |  |  |  |  |  |
| April | 7.3 | 4.6 | 8.4 | 3.0 | 3.0 | 7.3 | 5.3 | 8.0 | 4.2 | 4.6 |  |  |  |  |  |
| May | 11.1 | 9.5 | 9.8 | 10.4 | 9.5 | 11.2 | 9.8 | 10.5 | 11.4 | 10.4 |  |  |  |  |  |
| June | 13.9 | 14.4 | 15.1 | 12.1 | 14.2 | 14.7 | 14.1 | 15.0 | 12.4 | 14.6 |  |  |  |  |  |
| July | 16.0 | 17.4 | 16.3 | 14.8 | 15.3 | 16.4 | 16.9 | 17.1 | 14.8 | 16.7 |  |  |  |  |  |
| August | 15.0 | 15.2 | 14.6 | 14.3 | 13.9 | 15.8 | 15.7 | 15.3 | 14.4 | 15.6 |  |  |  |  |  |
| September | 11.9 | 9.6 | 11.6 | 9.6 | 9.4 | 12.7 | 10.9 | 12.7 | 10.8 | 11.4 |  |  |  |  |  |
| October | 1.9 | 4.1 | 6.5 | 3.6 | 4.8 | 4.1 | 5.8 | 8.3 | 4.4 | 7.0 |  |  |  |  |  |
| November | 3.5 | -4.3 | 3.0 | 2.5 | -1.6 | 4.5 | -2.1 | 5.2 | 2.4 | 1.8 |  |  |  |  |  |
| December | -6.8 | -15.3 | -1.3 | -7.7 | -6.3 | -4.1 | -10.9 | 1.5 | -5.5 | -1.6 |  |  |  |  |  |
| **Prec. (mm)** | Solør | | | | | Østfold | | | | |  | | | | |
| Month | 2009 | 2010 | 2011 | 2012 | Ø | 2009 | 2010 | 2011 | 2012 | Ø |  |  |  |  |  |
| January | - | - | - | - | 35 | 35 | 2 | - | - | 59 |  |  |  |  |  |
| February | - | - | - | - | 28 | 34 | 18 | - | - | 47 |  |  |  |  |  |
| March | - | - | - | - | 32 | 39 | 62 | - | - | 56 |  |  |  |  |  |
| April | 22 | 13 | 15 | 28 | 36 | 27 | 40 | 45 | 87 | 42 |  |  |  |  |  |
| May | 40 | 44 | 77 | 77 | 50 | 61 | 47 | 81 | 73 | 58 |  |  |  |  |  |
| June | 40 | 133 | 86 | 74 | 67 | 42 | 49 | 117 | 117 | 72 |  |  |  |  |  |
| July | 141 | 115 | 117 | 139 | 76 | 163 | 113 | 151 | 54 | 73 |  |  |  |  |  |
| August | 132 | 93 | 122 | 144 | 75 | 101 | 178 | 145 | 101 | 83 |  |  |  |  |  |
| September | 51 | 44 | 119 | 72 | 75 | 68 | 99 | 236 | 119 | 94 |  |  |  |  |  |
| October | 32 | 6 | 48 | 59 | 70 | 80 | 102 | 91 | 202 | 109 |  |  |  |  |  |
| November | - | - | - | - | 56 | 176 | 57 | - | - | 94 |  |  |  |  |  |
| December | - | - | - | - | 40 | 43 | 21 | - | - | 66 |  |  |  |  |  |
